# Reduced-dose vs full-dose direct oral anticoagulants for extended treatment of venous thromboembolism: a meta-analysis of randomized controlled trials

**DOI:** 10.1016/j.rpth.2025.102996

**Published:** 2025-08-05

**Authors:** Mushood Ahmed, Eeshal Zulfiqar, Hadiah Ashraf, Tallal Mushtaq Hashmi, Raheel Ahmed, Jamal S. Rana, Stephen J. Greene, Robert J. Mentz, Marat Fudim, Gregg C. Fonarow

**Affiliations:** 1 Rawalpindi Medical University, Rawalpindi, Pakistan; 2 Dow University of Health Sciences, Karachi, Pakistan; 3 Department of Cardiology, Royal Brompton Hospital, London, UK; 4 Division of Cardiology, National Heart and Lung Institute, Imperial College London, London, UK; 5 Division of Cardiology, Kaiser Permanente Northern California, Oakland, California, USA; 6 Division of Research, Kaiser Permanente Northern California, Oakland, California, USA; 7 Division of Cardiology, Duke University Medical Center, Durham, North Carolina, USA; 8 Division of Cardiology, Duke Clinical Research Institute, Durham, North Carolina, USA; 9 Division of Cardiology, Ahmanson-UCLA Cardiomyopathy Center, University of California Los Angeles, Los Angeles, California, USA

**Keywords:** anticoagulants, direct oral anticoagulants, factor Xa Inhibitors, meta-analysis, venous thromboembolism

## Abstract

**Background:**

Venous thromboembolism (VTE) is a major cause of cardiovascular morbidity and mortality globally. Although direct oral anticoagulants (DOACs) have improved extended VTE treatment, the optimal dose for balancing efficacy and safety remains unclear.

**Objectives:**

This systematic review and meta-analysis aimed to evaluate the efficacy and safety of reduced-dose DOACs vs full-dose regimens during extended anticoagulation for VTE.

**Methods:**

A literature search of PubMed, Embase, and Cochrane Library was performed up to April 2025 to identify randomized controlled trials (RCTs) comparing reduced-dose vs full-dose DOACs for extended VTE treatment in patients with or without cancer. Risk ratios (RR) and 95% CIs were estimated using a random-effects model. Primary outcomes were recurrent VTE and major or clinically relevant nonmajor bleeding. The secondary outcomes included major bleeding, clinically relevant nonmajor bleeding, all-cause mortality, and VTE-related mortality.

**Results:**

Five RCTs involving 8781 patients were included in the meta-analysis. The mean ± SD age of patients was 61.3 ± 13.4 years, and median follow-up duration was 12 months. Reduced-dose DOACs were comparable with full-dose regimens in preventing recurrent VTE (RR, 0.94; 95% CI, 0.68-1.29) and all-cause death (RR, 0.86; 95% CI, 0.63-1.17). However, reduced-dose DOACs significantly lowered the risk of major or clinically relevant nonmajor bleeding (RR, 0.71; 95% CI, 0.61-0.82), major bleeding (RR, 0.62; 95% CI, 0.42-0.92), and clinically relevant nonmajor bleeding (RR, 0.75; 95% CI, 0.63-0.88) compared with full-dose regimens. No significant subgroup differences were observed between cancer-associated and general VTE populations.

**Conclusion:**

Reduced-dose DOACs are as effective as full-dose regimens in preventing recurrent VTE and are associated with significantly lower bleeding risks. However, more RCTs with extended follow-up and focused inclusion of cancer patients are warranted to validate these findings.

## Essentials

• We assessed reduced-dose vs full-dose DOACs for extended VTE treatment.
• Reduced-dose DOACs were noninferior to full-dose therapy.
• Reduced-dose DOACs significantly lowered the risk of bleeding complications.
• There is no significant subgroup difference in cancer-associated vs general VTE populations.

## 1 Introduction

Venous thromboembolism (VTE), which includes deep vein thrombosis and pulmonary embolism, is a major global health burden and the third leading cause of cardiovascular mortality worldwide [1,2]. An estimated 10 million cases occur each year, contributing to >500,000 deaths in Europe and between 100,000 and 300,000 deaths in the United States [1,3]. Standard treatment involves anticoagulation therapy for a minimum of 3 months, effectively reducing the risk of recurrence during this period [4]. The risk of recurrence in patients with a first unprovoked VTE, despite having completed at least 3 months of anticoagulation, remains high, reaching approximately 10% within the first year after discontinuation [5]. While extending anticoagulation therapy beyond 3 to 6 months can significantly lower the risk of recurrent VTE, it also increases the risk of bleeding complications. As a result, extended-duration anticoagulation is reserved for patients at higher risk of recurrence [6].

While traditional vitamin K antagonists such as warfarin remain effective, they require regular laboratory monitoring and dose adjustments, making long-term management challenging [7]. In contrast, direct oral anticoagulants (DOACs) provide a more convenient alternative and have demonstrated a lower risk of recurrence in extended VTE treatment [8]. Given the need to minimize bleeding complications without compromising efficacy, reduced-dose DOACs have been evaluated as a viable option for extended anticoagulation. Randomized controlled trials (RCTs), including Apixaban after the Initial Management of Pulmonary Embolism and Deep Vein Thrombosis with First-Line Therapy–Extended Treatment (AMPLIFY-EXT) and Reduced-dosed Rivaroxaban in the Long-term Prevention of Recurrent Symptomatic Venous Thromboembolism (EINSTEIN CHOICE), have assessed the efficacy and safety of reduced-dose DOACs such as apixaban and rivaroxaban, demonstrating their ability to prevent VTE recurrence with a lower risk of bleeding [9,10]. In line with these findings, a meta-analysis by Vasanthamohan et al. [11] found that reduced-dose DOACs are as effective as full-dose anticoagulation in preventing VTE recurrences, with bleeding risks that are comparable with placebo or aspirin. However, while these findings support the use of reduced-dose DOACs, direct comparisons between full-dose and reduced-dose regimens in extended therapy remain limited, particularly in high-risk subgroups.

Recent trials, such as the Reduced Dose Versus Full-dose of Direct Oral Anticoagulant After Unprovoked Venous Thromboembolism (RENOVE) trial, have provided new insights into the efficacy and safety of reduced-dose DOACs for extended VTE treatment, highlighting the need for updated meta-analyses to guide clinical decision-making [12]. Additionally, recent studies have provided valuable data on specific patient subgroups, such as those with cancer-related VTE, which may influence the selection of appropriate anticoagulant therapies [13]. We, therefore, conducted a systematic review and meta-analysis to thoroughly assess the impact of reduced-dose DOACs on extended VTE treatment outcomes, including recurrence and bleeding risks. Moreover, a comprehensive evaluation that specifically accounts for the impact of underlying patient characteristics, such as the presence or absence of active cancer, remains crucial for guiding clinical practice. This meta-analysis aimed to address this gap by not only assessing the overall efficacy and safety of reduced-dose DOACs vs full-dose regimens but also by conducting a stratified meta-analysis based on whether trials enrolled patients with general VTE or cancer-associated VTE.

## 2 Methods

This systematic review and meta-analysis followed the Preferred Reporting Items for Systematic Reviews and Meta-Analyses guidelines [14]. The review protocol was registered with International Prospective Register of Systematic Reviews (CRD420251026816). No ethical approval or informed consent was required for this study.

### 2.1 Data sources and search strategy

Two authors (M.A. and H.A.) independently searched PubMed/MEDLINE, Embase, and the Cochrane Library to identify RCTs comparing reduced-dose vs full-dose DOACs for extended treatment of VTE. The search covered all available literature in the database from inception to April 3, 2025. Additional studies were identified by manually screening reference lists of eligible articles and relevant reviews. The search strategy included terms and medical subject headings such as (“DOAC” OR “direct oral anticoagulant” OR “apixaban” OR “rivaroxaban” OR “edoxaban” OR “dabigatran”) AND (“reduced dose” OR “low dose” OR “full dose” OR “standard dose”) AND (“venous thromboembolism” OR “VTE” OR “deep vein thrombosis” OR “pulmonary embolism”). The complete search strategies for each database are provided in Supplementary Table S1.

### 2.2 Study selection and eligibility criteria

Search results were imported into Mendeley Desktop, and duplicates were removed. Two authors (M.A. and H.A.) independently screened titles and abstracts for eligibility. Full texts of potentially relevant studies were assessed according to prespecified criteria. Any disagreements were resolved by discussion or consultation with a third reviewer (Z.A.N.). Eligible studies were RCTs with at least 2 months of follow-up, involving adult patients (age ≥18 years) with a history of VTE, comparing reduced-dose with full-dose DOACs during extended treatment (after an initial at least 3-6 months of full-dose therapy), and reporting at least 1 clinical outcome of interest. Primary outcomes included recurrent VTE and major bleeding or clinically relevant nonmajor bleeding. Secondary outcomes were major bleeding, clinically relevant nonmajor bleeding, all-cause mortality, and VTE-related mortality. The definitions used by each trial for reporting major bleeding and clinically relevant nonmajor bleeding are reported in Supplementary Table S2. Exclusion criteria included observational studies, case series, reviews, animal studies, and non–full-text articles. There were no language or data restrictions.

### 2.3 Data extraction

Two authors (H.A. and M.A.) independently extracted relevant data using a predesigned template. Extracted information included first author name or trial identifier, publication year, study design, sample size, follow-up duration, treatment regimens, outcome data, and baseline characteristics such as age, sex, sample size, duration of follow-up, cancer status, and renal function. The number of events and the total number of patients per group were recorded.

### 2.4 Bias assessment and certainty of evidence

The quality of included studies was assessed using the revised Cochrane risk of bias tool 2.0, evaluating domains such as the randomization process, deviations from intended interventions, missing outcome data, outcome measurement, and selection of reported outcomes [15]. Each study was rated as low risk, some concerns, or high risk. Certainty of evidence for each outcome was independently assessed by 2 authors (H.A. and M.A.) using the Grading of Recommendations Assessment, Development and Evaluation framework, which considers risk of bias, inconsistency, indirectness, imprecision, and publication bias. Evidence certainty was rated as high, moderate, low, or very low. Disagreements were resolved by a third reviewer (Z.A.N.).

### 2.5 Statistical analysis

Statistical analyses were performed using R software version 4.3.3 [16] with the “meta” [17] and “metasens” packages [18]. Pooled risk ratios (RRs) with 95% CIs were calculated using a random-effects model. Between-study heterogeneity was estimated using the Paule–Mandel method for τ^2^. The most extensive available follow-up data were used for each outcome. Forest plots were generated to illustrate pooled estimates. Heterogeneity was assessed using the chi-squared test and *I*^2^ statistic, with thresholds of <50%, 50% to 75%, and >75% interpreted as low, moderate, and high heterogeneity, respectively. Absolute risk differences were calculated by applying pooled RRs to the baseline risk of the full-dose DOAC groups. Summary of findings tables were generated using GRADEpro (https://gdt.gradepro.org/app/). A *P* value of < .05 was considered statistically significant. Subgroup analysis was conducted based on the trial population. Trials were classified as cancer-specific if they exclusively enrolled patients with cancer-associated VTE, and as general VTE trials if they included a broader VTE population with minimal inclusion of patients with cancer. Sensitivity analyses were performed using the leave-one-out method to assess the robustness of pooled estimates and identify any outlier studies.

## 3 Results

The systematic literature searches of databases identified 802 studies. The duplicate records (*n* = 258) were removed. Two investigators screened the remaining studies by reviewing their abstracts and titles, and 489 studies were excluded. This was followed by a review of the full texts of 55 records. A total of 5 studies [9,10,12,13,19] fulfilled the predetermined eligibility criteria and were ultimately included in the review (Supplementary Figure S1).

The trials reported data for 8781 patients (low dose: 4395 patients; full dose: 4386 patients). The mean ± SD age of patients was 61.8 ± 13.4 years, and 53.1% were male. Obesity (body mass index ≥ 30 kg/m^2^) prevalence ranged from 21% to 36%, and renal impairment (creatinine clearance ≤ 50 mL/min) was most frequent in Apixaban Cancer Associated Thrombosis (API-CAT). All trials enrolled adults (≥18 years) with symptomatic proximal deep vein thrombosis or pulmonary embolism who had completed initial anticoagulation (6-24 months). Reduced-dose DOAC regimens varied, with apixaban 2.5 mg twice daily used in AMPLIFY-EXT, Extending Venous Thromboembolism Secondary Prevention with Apixaban in Cancer Patients (EVE), and API-CAT trials, rivaroxaban 10 mg once daily in EINSTEIN CHOICE, and both options in RENOVE. Median follow-up was 12 months for all trials. Unprovoked VTE was common in AMPLIFY-EXT (≥90%) but less so in EINSTEIN CHOICE (∼40%) and RENOVE (63%-69%). Active cancer rates were negligible in AMPLIFY-EXT and EINSTEIN CHOICE (<3%), low in RENOVE (≈10%), and universal in API-CAT and EVE trials (Table 1). A low risk of bias was observed across all trials (Supplementary Figure S2). The Preferred Reporting Items for Systematic Reviews and Meta-Analyses checklist is reported in Supplementary Table S3.

**Table 1:** Baseline characteristics of the included studies and participants.

Trial		AMPLIFY-EXT 2013	EINSTEIN CHOICE 2017	EVE 2023	RENOVE 2025	API-CAT 2025
Inclusion criteria		Age ≥18 y, symptomatic proximal DVT or PE treated for 6-12 mo with full-dose anticoagulation, with clinical equipoise for stopping treatment.	Age ≥18 y, symptomatic proximal DVT or PE treated for 6-12 mo with full-dose anticoagulation.	Age ≥18 y with active cancer (evidence on imaging or treatment/surgery within the past 6 mo), had objectively confirmed acute VTE, and completed 6-12 mo of therapeutic anticoagulation before enrollment.	Age ≥18 y, symptomatic proximal DVT or PE treated for 6-24 mo with full-dose anticoagulation.	Patients with active cancer and symptomatic proximal DVT or PE who had completed at least 6 mo of treatment with a low-molecular-weight heparin, direct oral anticoagulant, or vitamin K antagonist.
Exclusion criteria		Contraindication to or definite indication for prolonged anticoagulation, hemoglobin < 90 g/L, platelet count < 100,000 mm^–3^, CrCl < 25 mL/min, and liver disease (elevated transaminases or bilirubin).	Contraindication to or definite indication for prolonged anticoagulation, CrCl < 30 mL/min, and liver disease with coagulopathy.	Anticoagulation for <6 or >12 mo before randomization; severe liver disease, active hepatitis, mechanical heart valves, bacterial endocarditis, or atrial fibrillation requiring stroke prevention; active bleeding or bleeding disorders; prior anticoagulant failure; pregnancy and nursing.	Contraindication to or definite indication for prolonged anticoagulation, high bleeding risk, renal insufficiency, on dual antiplatelet therapy or aspirin > 100 mg/d, active cancer within the previous 6 mo, pregnant, or a life expectancy of <12 mo.	Contraindication to or definite indication for prolonged anticoagulation, intracranial or intraocular bleeding within the previous 6 mo, systolic blood pressure > 180 mm Hg or diastolic blood pressure > 110 mm Hg, bacterial endocarditis, or a life expectancy of <12 mo.
Reduced-dose DOAC arm		Apixaban 2.5 mg twice daily	Rivaroxaban 10 mg once daily	Apixaban 2.5 mg twice daily	Apixaban 2.5 mg twice daily or rivaroxaban 10 mg once daily	Apixaban 2.5 mg twice daily
Full-dose anticoagulation arm		Apixaban 5 mg twice daily	Rivaroxaban 20 mg once daily	Apixaban 5 mg twice daily	Apixaban 5 mg twice daily or rivaroxaban 20 mg once daily	Apixaban 5.0 mg twice daily
Full-dose anticoagulation duration before randomization (mean)		6-12 mo	6-12 mo	6-12 mo	6-24 mo	≥6 mo
No anticoagulant arm		Placebo	Aspirin 81 mg once daily	NR	NR	NR
Median follow-up		12 mo	12 mo	12 mo	12 mo	12 mo
Sample size	Reduced dose	840	1127	179	1383	866
	Full dose	813	1107	181	1385	900
Age (y), mean ± SD	Reduced dose	56.6 ± 15.3	58.8 ± 14.7	63.6 ± 11	62.2 ± 14.3	67.2 ± 11.0
	Full dose	56.4 ± 15.6	57.9 ± 14.7	64.3 ± 10.7	63.1 ± 14.3	67.7 ± 11.4
Male (%)	Reduced dose	58	55	48.6	64.6	43.3
	Full dose	57.7	54.4	40.9	65.3	43.4
BMI ≥ 30 kg/m^2^ (%)	Reduced dose	NR	33.4	NR	31	22.4
	Full dose	NR	35.6	NR	30	21.3
CrCl ≤ 50 mL/min (*n*)	Reduced dose	48	51	NR	71	115
	Full dose	44	41	NR	91	127
Previous VTE (%)	Reduced dose	11.8	17.5	10.1	22.9	18.1
	Full dose	14.5	17.9	8.8	21.4	18.9
Active cancer (%)	Reduced dose	1.8	2.4	93.4	10.1	99.8
	Full dose	1.1	2.3	88	9.3	99.7
Unprovoked index VTE (%)	Reduced dose	93.2	42.6	NR	62.9	NR
	Full dose	90.7	39.8	NR	69	NR
Isolated DVT (%)	Reduced dose	64.8	50.1	49.2	14.6	22.7
	Full dose	64.8	51	50.8	13.7	26.1

AMPLIFY-EXT, Apixaban after the Initial Management of Pulmonary Embolism and Deep Vein Thrombosis with First-Line Therapy–Extended Treatment; API-CAT, Apixaban Cancer Associated Thrombosis; BMI, body mass index; CrCl, creatinine clearance; DOAC, direct oral anticoagulant; DVT, deep vein thrombosis; EINSTEIN CHOICE, Reduced-dosed Rivaroxaban in the Long-term Prevention of Recurrent Symptomatic Venous Thromboembolism; EVE, Extending Venous Thromboembolism Secondary Prevention with Apixaban in Cancer Patients; NR, not reported; PE, pulmonary embolism; RENOVE, Reduced Dose Versus Full-dose of Direct Oral Anticoagulant After Unprovoked Venous Thromboembolism; VTE, venous thromboembolism.

### 3.1 Outcomes

#### 3.1.1 Recurrent VTE

**Figure 1:**
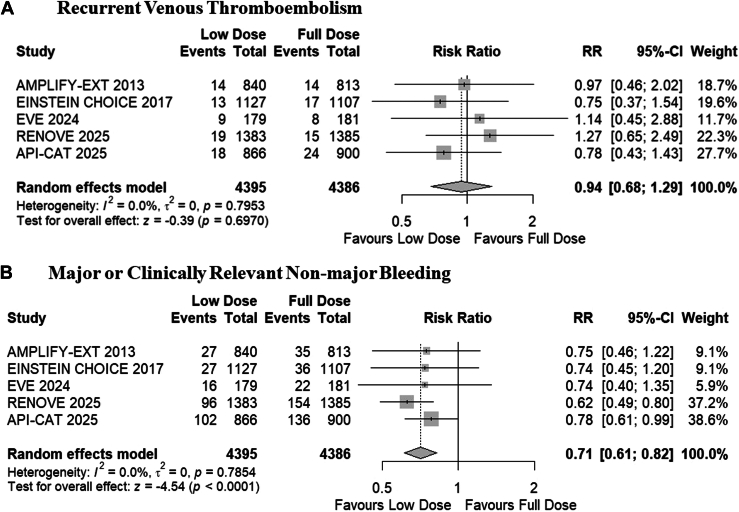
Forest plot for (A) recurrent venous thromboembolism and (B) major bleeding or clinically relevant nonmajor bleeding. AMPLIFY-EXT, Apixaban after the Initial Management of Pulmonary Embolism and Deep Vein Thrombosis with First-Line Therapy–Extended Treatment; API-CAT, Apixaban Cancer Associated Thrombosis; EINSTEIN CHOICE, Reduced-dosed Rivaroxaban in the Long-term Prevention of Recurrent Symptomatic Venous Thromboembolism; EVE trial, Extending Venous Thromboembolism Secondary Prevention with Apixaban in Cancer Patients; RENOVE, Reduced Dose Versus Full-dose of Direct Oral Anticoagulant After Unprovoked Venous Thromboembolism; RR, risk ratio.

All trials reported data for recurrent VTE. The pooled analysis demonstrated that the reduced dose of DOACs was comparable with full-dose DOACs for the risk of recurrent VTE (RR, 0.94; 95% CI, 0.68-1.29; *P* = .69; *I*^*2*^ *=* 0%; absolute risk difference, 1 [95% CI, 6 fewer to 5 more] per 1000 patients; moderate certainty; Figure 1A, Table 2). Subgroup analysis revealed no statistically significant difference in outcomes between trials enrolling patients with general VTE and those enrolling patients with cancer-associated VTE (*P* = .71; Supplementary Figure S3).

**Table 2:** Summary of findings table.

Outcome	*N* patients (studies)	RR (95% CI)	Absolute effects (95% CI)			Certainty	What happens
			Full dose of DOACs	Reduced dose of DOACs	Difference		
Recurrent VTE	8781 (5 RCTs)	0.94 (0.68-1.29)	18 per 1000	17 per 1000 (12-23)	1 fewer per 1000 (from 6 fewer to 5 more)	⨁⨁⨁◯ Moderate	Reduced-dose DOACs likely result in little to no difference in recurrent VTE.
Major bleeding or clinically relevant nonmajor bleeding	8781 (5 RCTs)	0.71 (0.61-0.82)	87 per 1000	62 per 1000 (53-72)	25 fewer per 1000 (from 34 fewer to 16 fewer)	⨁⨁⨁⨁ High	Reduced-dose DOACs reduce major bleeding or clinically relevant nonmajor bleeding.
Major bleeding	8781 (5 RCTs)	0.62 (0.42-0.92)	20 per 1000	12 per 1000 (8-18)	8 fewer per 1000 (from 13 fewer to 3 fewer)	⨁⨁⨁⨁ High	Reduced-dose DOACs reduce major bleeding.
Clinically relevant nonmajor bleeding	8781 (5 RCTs)	0.75 (0.63-0.88)	70 per 1000	53 per 1000 (44-62)	17 fewer per 1000 (from 26 fewer to 8 fewer)	⨁⨁⨁⨁ High	Reduced-dose DOACs reduce clinically relevant nonmajor bleeding.
All-cause death	8781 (5 RCTs)	0.86 (0.63-1.17)	58 per 1000	50 per 1000 (37-68)	8 fewer per 1000 (from 22 fewer to 10 more)	⨁⨁⨁◯ Moderate	Reduced-dose DOACs likely result in little to no difference in all-cause death.
VTE-related deaths	8421 (4 RCTs)	0.70 (0.17-2.89)	1 per 1000	1 per 1000 (0-3)	0 fewer per 1000 (from 1 fewer to 2 more)	⨁⨁⨁◯ Moderate	Reduced-dose DOACs likely result in little to no difference in VTE-related deaths.

DOAC, direct oral anticoagulant; RCT, randomized controlled trial; RR, risk ratio; VTE, venous thromboembolism.

#### 3.1.2 Major bleeding or clinically relevant nonmajor bleeding

All trials reported data for major bleeding or clinically relevant nonmajor bleeding. The pooled analysis demonstrated that the reduced dose of DOACs was associated with a significantly reduced risk of major bleeding or clinically relevant nonmajor bleeding compared with full-dose DOACs (RR, 0.71; 95% CI, 0.61-0.82; *P* ≤ .01; *I*^*2*^ *=* 0%; absolute risk difference, 25 [95% CI, 34-16] fewer per 1000 patients; high certainty; Figure 1B). The subgroup analysis indicated no statistically significant difference between the general VTE population and the cancer-associated VTE population (*P* = .30; Supplementary Figure S4).

#### 3.1.3 Major bleeding

**Figure 2:**
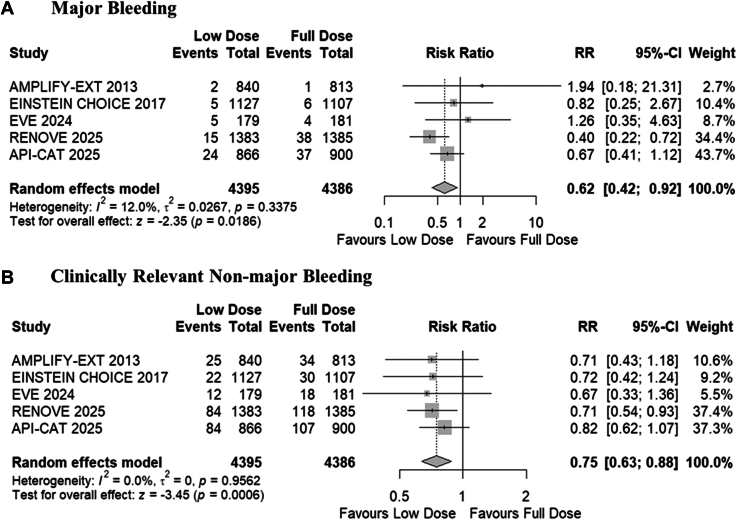
Forest plots for (A) major bleeding and (B) clinically relevant nonmajor bleeding. AMPLIFY-EXT, Apixaban after the Initial Management of Pulmonary Embolism and Deep Vein Thrombosis with First-Line Therapy–Extended Treatment; API-CAT, Apixaban Cancer Associated Thrombosis; EINSTEIN CHOICE, Reduced-dosed Rivaroxaban in the Long-term Prevention of Recurrent Symptomatic Venous Thromboembolism; EVE, Extending Venous Thromboembolism Secondary Prevention with Apixaban in Cancer Patients; RENOVE, Reduced Dose Versus Full-dose of Direct Oral Anticoagulant After Unprovoked Venous Thromboembolism; RR, risk ratio.

All trials reported data for major bleeding. The pooled analysis demonstrated that the reduced dose of DOACs was associated with a significantly reduced risk of major bleeding compared with full-dose DOACs (RR, 0.62; 95% CI, 0.42-0.92; *P* = .01; *I*^*2*^ *=* 12%; absolute risk difference, 8 [95% CI, 13-3] fewer per 1000 patients; high certainty; Figure 2A). No significant subgroup difference was observed between cancer-associated and general VTE populations (*P* = .46; Supplementary Figure S5).

#### 3.1.4 Clinically relevant nonmajor bleeding

All trials reported data for clinically relevant nonmajor bleeding. The pooled analysis demonstrated that the reduced dose of DOACs was associated with a significantly reduced risk of clinically relevant nonmajor bleeding compared with full-dose DOACs (RR, 0.75; 95% CI, 0.63-0.88; *P* ≤ .01; *I*^*2*^ *=* 0%; absolute risk difference, 17 [95% CI, 26-8] fewer per 1000 patients; high certainty; Figure 2B). No significant subgroup difference was observed between cancer-associated and general VTE populations (*P* = .52; Supplementary Figure S6).

#### 3.1.5 Deaths

**Figure 3:**
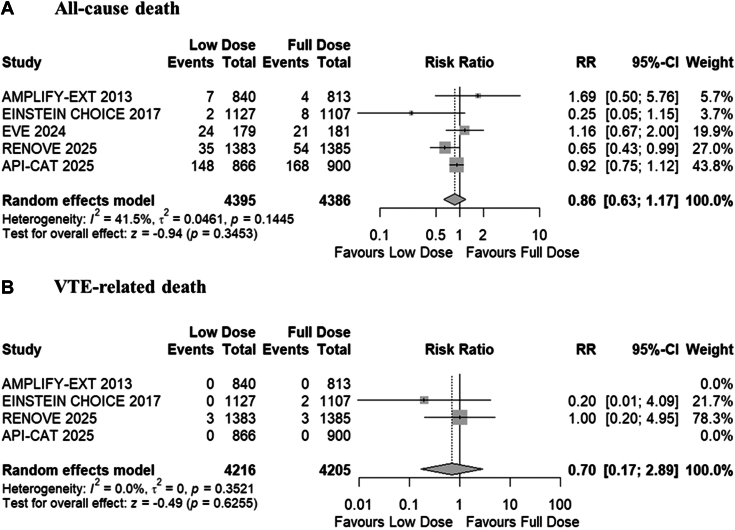
Forest plots for (A) all-cause death, and (B) venous thromboembolism (VTE)-related deaths. AMPLIFY-EXT, Apixaban after the Initial Management of Pulmonary Embolism and Deep Vein Thrombosis with First-Line Therapy–Extended Treatment; API-CAT, Apixaban Cancer Associated Thrombosis; EINSTEIN CHOICE, Reduced-dosed Rivaroxaban in the Long-term Prevention of Recurrent Symptomatic Venous Thromboembolism; EVE, Extending Venous Thromboembolism Secondary Prevention with Apixaban in Cancer Patients; RENOVE, Reduced Dose Versus Full-dose of Direct Oral Anticoagulant After Unprovoked Venous Thromboembolism; RR, risk ratio.

The pooled analysis demonstrated a comparable rate of all-cause death (RR, 0.86; 95% CI, 0.63-1.17; *P* = .34; *I*^*2*^ *=* 41.5%; moderate certainty; Figure 3A) and VTE-related deaths (RR, 0.70; 95% CI, 0.17-2.89; *P* = .63; *I*^*2*^ *=* 0%; moderate certainty; Figure 3B) across the 2 groups. Subgroup analysis showed comparable results for cancer-associated and general VTE groups (Supplementary Figures S7 and S8). No outlier study was identified in the leave-one-out sensitivity analysis for all outcomes (Supplementary Figures S9–S14).

## 4 Discussion

In this meta-analysis of 8781 patients, we evaluated the efficacy and safety of reduced-dose DOACs for extended anticoagulation in VTE. This meta-analysis demonstrated with moderate certainty evidence that reduced-dose DOACs were as effective as full-dose treatment in preventing recurrent VTE, while significantly lowering the risk of major bleeding and clinically relevant nonmajor bleeding (high certainty evidence). Additionally, rates of all-cause mortality and VTE-related mortality were comparable between the 2 groups. These results help to strengthen the suggestion that dose reduction does not compromise the efficacy of preventing recurrent VTE while offering a safer alternative with reduced bleeding risks.

Our findings extend the current literature regarding the safety and efficacy of reduced vs full-dose DOACs following VTE. A prior meta-analysis by Vasanthamohan et al. [11] also demonstrated similar recurrence rates with reduced-dose DOACs, though their sample size was substantially smaller. The comparable efficacy in preventing recurrent VTE is clinically significant, especially in patients who are at higher risk of bleeding, such as those with cancer, renal impairment, or older age [[20], [21], [22]]. In addition, for individuals at an increased risk of recurrence, maintaining protection without increasing bleeding risk offers a crucial advantage in long-term VTE management [6]. Several clinical trials have provided robust evidence supporting our findings. The AMPLIFY-EXT trial demonstrated that both 2.5 mg and 5 mg doses of apixaban significantly reduced the risk of recurrent VTE compared with placebo, with recurrence rates of 1.7% for both doses [9]. Similarly, the EINSTEIN CHOICE trial found that rivaroxaban at doses of 10 mg and 20 mg daily significantly lowered the risk of recurrent VTE compared with aspirin, with recurrence rates of 1.2% for 10 mg vs 1.5% for 20 mg [10]. More recently, the Reduced Dose Versus Full-dose of Direct Oral Anticoagulant After Unprovoked Venous Thromboembolism (RENOVE) trial directly compared reduced-dose and full-dose DOAC regimens for extended anticoagulation in patients at high risk of VTE recurrence [12]. Although the reduced-dose regimen did not meet the predefined noninferiority criteria, the absolute recurrence rates were low in both groups. One plausible explanation for the lower-than-expected recurrence rates in RENOVE could be the high adherence to treatment observed throughout follow-up, with more than two-thirds of patients remaining on therapy and a treatment discontinuation rate of only 16% at 5 years. This adherence was comparable with or better than previous trials such as AMPLIFY-EXT and EINSTEIN CHOICE, where treatment discontinuation rates were 14.7% and 12.5% at 1 year, respectively. These findings suggest that in a real-world setting, where adherence may be lower, the recurrence risk with reduced-dose DOACs could be more pronounced.

Building on our findings on the general VTE population, it is imperative to consider their application in patients with active cancer who are at a considerably higher risk for recurrent VTE [20]. VTE is the most frequent thrombotic complication in patients with cancer, with the risk estimated to be approximately 12 times higher than in their noncancer counterparts [23,24]. This elevated risk is attributed to a combination of factors, including chemotherapy, radiotherapy, surgery, and patient-specific factors such as immobility and advanced age [25,26]. Current guidelines recommend anticoagulation for a minimum duration of 6 months, with extended therapy advised for as long as the cancer remains active or treatment continues, provided the benefits outweigh the ongoing risk of bleeding complications [[26], [27], [28]]. While low-molecular-weight heparin has been the standard treatment for the past decade, recent evidence suggests that DOACs may offer comparable efficacy in preventing recurrent VTE. In their meta-analysis, Li et al. [29] demonstrated DOACs to be more effective in reducing VTE recurrences, though this benefit was associated with an increased risk of bleeding events. Given these considerations, the potential role of reduced-dose DOACs in cancer patients becomes increasingly relevant and has been explored in recent research, such as the API-CAT trial [13]. Our subgroup analysis, incorporating cancer-specific trials such as API-CAT and EVE, offers important insights into the use of reduced-dose DOACs for cancer-associated VTE. While our meta-analysis found comparable efficacy with full-dose regimens and a lower bleeding risk, these findings must be interpreted with caution in cancer patients who face elevated risks of both recurrence and bleeding. Current American Society of Hematology and European Society of Cardiology guidelines increasingly support DOAC use in selected cancer patients, especially those at low bleeding risk [28,30,31]. The API-CAT trial, in particular, demonstrated that reduced-dose apixaban was noninferior in preventing recurrence while reducing bleeding. These results support the use of reduced-dose DOACs in carefully selected cancer patients requiring extended therapy. However, clinical decisions should be individualized, considering cancer type, treatment status, and bleeding risk. Further dedicated trials are needed to confirm these findings across diverse cancer populations. Furthermore, a study by Vichaidit et al. [32] evaluating cancer-associated VTE patients provides additional insights. They found that, while the incidence of recurrent VTE was numerically higher in the reduced-dose group (5%) compared with the full-dose group (2.4%), the difference was not statistically significant [32]. These findings suggest that while reduced-dose anticoagulation may reduce bleeding risk, it could potentially compromise the efficacy in preventing VTE recurrence, particularly in cancer patients, where the balance between bleeding and thrombosis is critical.

Our analysis also demonstrated a significantly lower risk of major bleeding and clinically relevant nonmajor bleeding. This finding contrasts with the previous meta-analysis, which reported no significant differences in bleeding risks between reduced-dose DOACs and full-dose anticoagulation in extended VTE treatment [11]. This discrepancy may stem from differences in study populations, follow-up durations, and our larger sample size, which likely provided greater statistical power to detect differences in bleeding outcomes. All included studies in our analysis adhered to similar definitions for major bleeding and clinically relevant nonmajor bleeding, primarily based on the International Society on Thrombosis and Haemostasis criteria [33]. While we observed no significant difference in the rates of all-cause mortality and VTE-related mortality between the 2 groups, evidence from Ahmed et al. [34] suggests a potential benefit of transitioning to low-dose DOACs regarding these outcomes. Specifically, they reported a significant reduction in all-cause mortality among patients who switched to low-dose DOACs, despite no significant association with VTE recurrence or major bleeding. The lack of differences in VTE recurrence or major bleeding between the 2 groups suggests that the observed reduction in mortality may not be directly related to improvements in VTE outcomes. Rather, it could reflect factors such as better patient tolerance to lower doses, a reduction in treatment-related complications, or improved adherence, which might influence long-term survival. Lower doses may also reduce the risk of adverse events, such as major bleeding, thereby contributing to better overall health outcomes. Furthermore, the relatively low incidence of adverse outcomes (death, major bleeding, or life-threatening bleeding) in the included studies may reflect the younger age and fewer comorbidities in the VTE population compared with patients with other indications for DOAC therapy. Given these variations, future studies with longer follow-up and more diverse populations are necessary to confirm these findings.

The duration of initial full-dose anticoagulation is a critical factor when considering transition to extended therapy. In our meta-analysis, included trials enrolled patients who had completed 6 to 24 months of prior anticoagulation. For example, AMPLIFY-EXT, EVE, and EINSTEIN CHOICE typically enrolled patients after 6 to 12 months, while RENOVE allowed inclusion up to 24 months. This variability is important, as patients with longer initial treatment may have a lower residual risk of recurrence upon stepping down to a reduced dose. Understanding the timing of dose reduction in relation to initial treatment duration is essential for risk stratification. Future studies should consistently report the mean or median duration of prior full-dose therapy to enable better comparison across trials and support individualized decisions on when to transition to reduced-dose anticoagulation.

### 4.1 Limitations

This meta-analysis has several limitations that should be considered when interpreting the results. First, our analysis included a limited number of trials, representing only 2 of the 4 approved DOACs for acute VTE treatment. Second, the control groups varied between studies, with some using a placebo and others using aspirin, which may influence the comparability of outcomes. Third, all included studies followed patients for only 1 year after acute VTE treatment; trials with long-term follow-up are necessary to validate our findings and draw more definitive conclusions regarding the long-term impact of reduced-dose DOACs. Lastly, race, ethnicity, and socioeconomic factors were not consistently reported across the included studies, which limits the generalizability of our findings to diverse populations. These factors, as well as variations in comorbidities and healthcare access, could have influenced treatment outcomes and should be addressed in future research. Additional well-powered RCTs would enhance the level of evidence.

## 5 Conclusion

This meta-analysis provides evidence supporting the efficacy and safety of reduced-dose DOACs for extended-phase anticoagulation in VTE. Reduced-dose regimens were found to be as effective as full-dose treatments in preventing recurrent VTE while significantly lowering the risk of major bleeding and clinically relevant nonmajor bleeding. These findings suggest that reduced-dose DOACs may offer a safer alternative for patients at a higher bleeding risk without compromising protection from recurrent VTE events. While our findings support the use of reduced-dose DOACs for extended VTE treatment up to 12 months, evidence beyond this period is limited. With a median follow-up of only 12 months across trials, long-term efficacy and safety remain uncertain, highlighting the need for studies with extended follow-up to determine optimal treatment duration. In cancer-associated VTE, our subgroup analysis indicates that reduced-dose DOACs may balance efficacy and bleeding risk effectively, aligning with current guideline trends. However, due to the complexity of cancer-related thrombosis, treatment decisions should be individualized. Further research is essential to clarify the role of reduced-dose regimens in specific cancer populations.
